# Abnormalities of Oocyte Maturation: Mechanisms and Implications

**DOI:** 10.3390/ijms252212197

**Published:** 2024-11-13

**Authors:** Giorgio Maria Baldini, Dario Lot, Antonio Malvasi, Antonio Simone Laganà, Antonella Vimercati, Miriam Dellino, Ettore Cicinelli, Domenico Baldini, Giuseppe Trojano

**Affiliations:** 1 Obstetrics and Gynecology Unit, Department of Biomedical Sciences and Human Oncology, University of Bari “Aldo Moro”, 70121 Bari, Italy; gbaldini97@gmail.com (G.M.B.); antoniomalvasi@gmail.com (A.M.); antonella.vimercati@uniba.it (A.V.); miriam.dellino@uniba.it (M.D.); ettore.cicinelli@uniba.it (E.C.); 2 IVF Center, Momo Fertilife, 76011 Bisceglie, Italy; danieleferrimomo@gmail.com; 3 Unit of Obstetrics and Gynecology “Paolo Giacone” Hospital, Department of Health Promotion, Mother and Child Care, Internal Medicine and Medical Specialities (PROMISE), University of Palermo, 90135 Palermo, Italy; antoniosimone.lagana@unipa.it; 4 Department of Maternal and Child Health, Madonna delle Grazie Hospital, 75010 Matera, Italy; giuseppe.trojano@asmbasilicata.it

**Keywords:** assisted reproductive technologies (ART), oocyte maturation, in vitro fertilization (IVF), abnormalities oocyte maturation

## Abstract

The elucidation of oocyte maturation mechanisms is paramount for advancing embryo development within the scope of assisted reproductive technologies (ART). Both cytoplasmic and nuclear maturation represent intricate processes governed by tightly regulated cellular pathways, which are essential for ensuring the oocyte’s competence for fertilization and subsequent embryogenesis. A comprehensive grasp of these mechanisms is vital, as the maturation stage of the oocyte significantly influences chromosomal integrity, spindle formation, and its ability to support the initial stages of embryonic development. By leveraging this knowledge, we can enhance in vitro fertilization (IVF) protocols, refining ovarian stimulation regimens and culture conditions to improve oocyte quality. This, in turn, has the potential to boost pregnancy rates and outcomes. Further research in this area will contribute to the development of novel interventions that aim to increase the efficacy of preimplantation embryonic development, offering new opportunities for individuals undergoing fertility treatments.

## 1. Introduction

Despite significant advances in understanding the process of oocyte growth and maturation, we cannot yet claim to have acquired comprehensive knowledge on the subject. Numerous phases of these processes remain only partially elucidated, and this gap is reflected in our ability to obtain qualitatively competent oocytes, which are crucial for the effectiveness and optimization of assisted reproductive technologies (ART) protocols. The retrieval of incompetent oocytes during oocyte collection is a relatively common phenomenon.

This review thoroughly examines the known factors that play a crucial role in oocyte maturation, considering both the nuclear and cytoplasmic aspects. Additionally, it will assess factors that, while not directly related to these two phases, may still influence oocyte competence. Oocyte development and the formation of primordial follicles are fundamental processes in female reproductive biology, intrinsically linked to reproductive capacity and fertility. These processes begin early in embryonic life and involve a complex series of cellular and molecular events determining the future ovarian reserve. Understanding these mechanisms is essential for outlining the physiological basis of human reproduction and for identifying potential therapeutic interventions in cases of infertility or other reproductive dysfunctions.

Understanding the ovarian reserve and the mechanisms underlying the maturation and recruitment of primordial follicles and their precursors is crucial to managing patients with compromised ovarian reserves. Recent scientific studies [1] have demonstrated the presence of primordial follicle complexes in the ovarian cortex of patients with very low levels of anti-Müllerian hormone (AMH). Identifying molecules and mechanisms that promote the differentiation of these primordial follicles into mature follicles could represent a significant therapeutic strategy for patients with inadequate ovarian response (poor responders), potentially decreasing reliance on egg donation.

The formation of oocytes begins with the differentiation of primordial germ cells [2], which migrate towards the developing gonads, where they transform into oogonia [3]. The transition from oogonia to oocytes is marked by entry into meiosis [4], which imparts the genetic characteristics necessary for fertilization and embryonic development to the oocytes [5]. In humans, the onset of meiosis occurs between the 11th and 12th weeks of gestation [6], when the oogonia enter prophase I and arrest in the diplotene subphase, thus forming oocytes [7]. During prophase I, homologous recombination and the pairing of homologous chromosomes occur [8]. Recombination enables the exchange of genetic material through crossing-over, initiated by the formation of double-strand breaks (DSBs), which subsequently require repair [9]. To facilitate this process, the synaptonemal complex (SC) assembles along each chromatid, ensuring cohesion and promoting synapsis [10]. The regulation of DSBs is crucial: an excessive number could threaten genomic integrity, while an insufficient number may impede successful recombination. Synapsis must be maintained until recombination is fully accomplished, ensuring proper chromosomal alignment and minimizing errors in DSB repair [11].

The retention of oocytes in the diplotene stage of prophase I is a defining characteristic that persists until they are stimulated to restart meiosis in the postnatal ovarian cycle. During this arrest period, oocytes are particularly vulnerable to various regulatory processes, including survival and apoptotic signals [12], determining their future capacity to mature and ovulate [13]. The balance between these signals is critical for forming the ovarian reserve, reflecting the overall quantity of primordial follicles present at birth [14].

The progression to the diplotene stage requires intricate interactions with somatic cells, particularly granulosa cells [15]. These cells organize around the oocyte to form a complex structure enclosed by a basement membrane, giving rise to the primordial follicle [16]. The formation of primordial follicles is a critical event that establishes the ovarian reserve, determining a woman’s reproductive potential throughout her life [17]. During this phase, the breakdown of germ cell cysts and the formation of cellular connections between oocytes and granulosa cells are key processes [18]. The regulation of these events is mediated by various factors, including apoptotic proteins and anti-apoptotic factors that modulate oocyte survival during follicle formation [19]. The communication between oocytes and granulosa cells is essential for maintaining oocyte quality and regulating the process of folliculogenesis [20]. This molecular dialogue is mediated by a series of paracrine and autocrine signals that ensure the oocyte’s survival and its ability to mature into a fully competent oocyte ready for fertilization [21]. Growth factors are crucial for maintaining the integrity of communication between oocytes and granulosa cells [22]. These polypeptides promote oocyte survival and regulate the cell cycle of granulosa cells, thereby facilitating follicle formation and growth [23]. Although the importance of these processes is well established, many elements of the molecular regulation of human folliculogenesis are still not fully understood. The variances between animal models and human systems pose significant challenges in applying laboratory findings to clinical settings. Nonetheless, research continues to investigate the mechanisms that govern the communication between oocytes and somatic cells, aiming to enhance therapeutic approaches for infertility and to protect women’s fertility.

## 2. Oocyte Maturation—Nuclear Maturation

The molecular mechanisms underlying the various stages of oocyte maturation, the transition from maternal to embryonic control, and the early phases of pre-embryonic development are regulated by distinct key genes. The cytoplasmic and nuclear maturation of the oocyte during pre-ovulatory development can be considered separate entities, and some researchers [24] have demonstrated that a well-balanced energetic metabolism in oocytes significantly impacts both and further embryonic developmental competence. Cytoplasmic maturation and the acquisition of RNA and protein reserves dominate the development of oocytes from the primordial to the pre-ovulatory stages [25].

The initiation of nuclear maturation is morphologically characterized by the dissolution of the oocyte’s nuclear envelope, commonly referred to as “germinal vesicle breakdown” (GVBD) [26], which is triggered by the mid-cycle peak of luteinizing hormone (LH) [27]. In vitro, this event is associated with decreased intracellular cAMP concentrations. Cyclic nucleotides, cAMP and cGMP, are key molecules in the early events that induce meiosis resumption triggered by LH [28].

The decrease in cAMP [29] within the oocyte is a result of several parallel or consecutive processes involving an increase in phosphodiesterase (PDE) activity, the enzyme responsible for breaking the phosphodiester bonds of cyclic nucleotides [30], and a reduction in the transport of substances from the surrounding somatic cells. Different forms of PDE have been identified in various tissues, particularly at the follicular level, where a compartmentalized organization has been observed, with type 3 PDE in oocytes and type 4 PDE in granulosa cells [31].

Petersen TS et al. [32] conducted analyses of mRNA microarray data from follicles and granulosa cells (GC), combined RT-PCR analysis, and enzymatic activity assessments in GC, as well as immunohistochemical analysis of ovarian sections and studies on the effects of PDE inhibitors. These investigations revealed that the activity of this enzyme is fundamental in the control of oocyte maturation. The central role of phosphorylation in the regulation of meiosis is underscored by the numerous protein kinases involved in oocyte maturation, including protein kinase A (PKA) and its oocyte-specific anchoring protein (AKAP), which is phosphorylated in oocytes resuming meiosis [33], as well as G protein kinase (PKG) and protein kinase C (PKC), which can be activated by Ca^2+^, phospholipids, and diacylglycerol [34]. Other proteins implicated in this complex process include protein phosphatase-39 (PP39) and cyclin-dependent protein kinase 2 (cdc2) [35]. Changes in cyclic nucleotide and purine levels likely influence phosphorylation events.

The pre-ovulatory peak of LH leads to the activation of adenylate cyclase C (AC) and PKC, along with a reduction in the activity of the vitamin D binding protein (VDBP) in somatic cells. Additionally, homologous gap junctions in the cumulus mass disappear and due to disrupted gap junctions and reduced cGMP levels, the transfer of these compounds into the oocyte decreases. Lower cGMP levels within the oocyte result in the cessation of PDE (phosphodiesterase-E) activity inhibition, reducing cAMP levels. PKA’s activity decreases, and PKG and PKC’s activity may also diminish. This initiates a cascade of phosphorylation and dephosphorylation events, leading to changes in transcriptional patterns, translation, and the activity of various proteins [36].

In a study published by Wang et al. [37], a substrate for PKA was developed that allows the monitoring of PKA activity in live oocytes. The study revealed that during progesterone-induced oocyte maturation, PKA was rapidly inactivated within 30 min of progesterone addition and maintained this inactive state throughout the maturation process. Furthermore, the induced activation of endogenous PKA had different effects depending on the timing of reactivation. Reactivation at any time before germinal vesicle breakdown inhibited GVBD. In contrast, reactivation post-GVBD did not interfere with the transition from meiosis I to meiosis II or the arrest in metaphase II. These findings provide initial evidence of PKA restriction and permissive phases in oocyte maturation, highlighting the critical importance of the temporal regulation of PKA activity during this complex biological process.

This phase and many subsequent phases of meiosis are controlled by the maturation-promoting factor (MPF) [25]. Although the constituents of MPF, specifically the p34cdc2 kinase and cyclin B, are also present in mitotically dividing cells, the regulation of MPF activity differs in meiotically dividing cells. A specific oocyte kinase, c-mos, plays a crucial role in upregulating MPF activity during the various stages of final oocyte maturation. Proper functioning of the c-mos-MPF system is associated with critical characteristics of the late stages of oocyte maturation, such as the resumption of meiotic maturation, the inhibition of DNA replication between meiosis I and II, and the maintenance of oocyte arrest in metaphase II until fertilization.

Finally, the degradation of c-mos and active MPF after fertilization enables the initiation of mitotic cell division in the pre-embryo. The early cell divisions of the human pre-embryo are still controlled by mRNA and proteins inherited from the mother. Zygotic gene expression begins between the 4-cell and 8-cell stages, after which the pre-embryo utilizes its genes. Some early genes expressed in the human pre-embryo encode proteins associated with cell division, extracellular growth modulation signals, and implantation-associated factors [38]. Table 1 lists the factors that can influence nuclear maturation.

## 3. Oocyte Maturation—Cytoplasmic Maturation

The regulation of human oocyte maturation appears to be a multifactorial process involving several signaling transduction pathways. Various research groups [25,39] have identified several downstream mechanisms that affect FSH and LH. Some of the identified mechanisms include the regulation of cAMP/cGMP levels in oocytes [40], mediated by C-type natriuretic peptide (CNP), the effects of EGF-related peptides such as amphiregulin (AREG) and/or epiregulin (EREG) [41], the influence of members of the TGF-beta family, including growth differentiation factor 9 (GDF9) and bone morphogenetic protein 15 (BMP15) [42], activin/inhibin, the meiosis-activating sterol in follicular fluid (FF-MAS) [43,44], midkine growth factor (MDK) [45], and many others. However, it is still unclear to what extent these pathways and mechanisms are active in humans in vivo.

A prospective study [39] included 50 women undergoing infertility treatment following a standard protocol with antagonists. The substances and signaling pathways that might influence human oocyte maturation in follicular fluid (FF) and granulosa cells (GC) were analyzed at five time points during the final maturation of follicles: CNP, EGF family, inhibin-A, inhibin-B, activin, FF-MAS, MDK, GDF9, and BMP15. All substances and signaling pathways assessed are potentially active in regulating human oocyte maturation in vivo, except for the GDF9/BMP15 signaling pathway. Notably, AREG, inhibins, and MDK were significantly upregulated during the first 12–17 h after the initiation of final follicle maturation, with considerably higher concentrations than previously reported. The genes regulating FF-MAS synthesis and metabolism were also significantly controlled to favor accumulation during the first 12–17 h. In contrast, CNP concentrations were low and did not vary during the final maturation process of the follicle. The concentrations of GDF9 and BMP15 were much lower than those reported in small antral follicles, suggesting a reduced influence of these substances.

A research group [46] examined oocyte-cumulus complexes collected from three patient groups: the first group included women with unstimulated ovaries undergoing surgery; the second group involved patients with multifollicular development induced by FSH and LH without an ovulatory dose of hCG; and the third group comprised oocyte-cumulus complexes retrieved following full ovarian stimulation with FSH, LH, and an ovulatory dose of hCG. The comparison of spontaneous resumption of meiosis and metaphase II oocytes among the groups revealed significant differences between unstimulated and stimulated ovaries after 24 and 48 h of culture. The administration of hCG accelerated the maturation percentage at the 24 h mark. Further incubations of unstimulated oocyte-cumulus complexes with EGF and IGF-I significantly increased the rate of metaphase II oocytes after 24 and 48 h of culture. The growth factors EGF and IGF-I may enhance the spontaneous maturation of immature human oocytes. Because spontaneous maturation is primarily observed when follicles have been exposed to pharmacological doses of hMG, it is suggested that increased levels of FSH within the follicle coincide with the generation of a positive signal necessary to complete oocyte maturation in humans. This signal may be related to the dynamics of growth factors within the follicle.

The potential use of EGF as an inducer of cytoplasmic and nuclear maturation was investigated by the same research group [47] in women undergoing ART with more than ten oocytes retrieved after oocyte pick-up (OPU). Oocytes from 17 high-responder patients were randomly assigned to one of three treatment groups at the time of retrieval: control without EGF (n = 93), EGF at 1.0 ng/mL (n = 92), and EGF at 10.0 ng/mL (n = 77) for 6 h before fertilization. The observed fertilization rates were 54.6%, 59.0%, and 46.1%, respectively, suggesting that EGF is ineffective in this maturation phase after the specified exposure period. The development of embryos was further analyzed by considering the morphological appearance of embryos under a microscope and the number of blastomeres developed 48 h after fertilization—no significant differences among the groups regarding the number of developed blastomeres were found. However, embryos derived from oocytes treated with 10 ng/mL of EGF exhibited poorer morphological appearance under the microscope. It was concluded that a 6 h incubation with EGF does not seem to influence cytoplasmic maturation in oocytes obtained after treatment.

To investigate the role of insulin-like growth factors (IGF) in human ovulation, a research group evaluated the concentrations of insulin-like growth factor binding protein 1 (IGFBP-1) in follicular fluid (FF) [48]. The concentrations of IGFBP-1 in the FF of 15 women undergoing ART treatment were measured and correlated with levels of 17beta-estradiol (E2), progesterone, and androstenedione in the FF. IGFBP-1 levels in the FF positively correlated with E2 and progesterone levels. No correlation was found between IGFBP-1 and androstenedione levels in the FF. IGFBP-1 concentrations were significantly higher in FF containing mature oocytes than those containing immature oocytes, while IGFBP-3 in the FF tended to decrease with oocyte maturation. These results suggest that IGF may play a significant role in human pre-ovulatory processes and that IGFBP-1 could be a valuable biochemical marker for assessing oocyte maturation.

A more recent publication [49] focused on investigating the association between levels of IGF, IGFBP, and pregnancy-associated plasma protein A (PAPP-A) in the follicular fluid (FF) and the quality of subsequent embryonic development from in vitro fertilized oocytes collected from the same follicle. FF samples and corresponding mature oocytes were collected and analyzed during oocyte retrieval. Using enzyme-linked immunosorbent assay (ELISA), IGF-I, IGF-II, IGFBP-1, IGFBP-3, IGFBP-4, and PAPP-A levels in the FF were determined. The progesterone secretion capacity of granulosa cells (GC) was measured using radioimmunoassay (RIA). Results showed that levels of IGF-II, IGFBP-3, and IGFBP-4 in the FF at the time of oocyte recovery were significantly correlated with embryonic classification on day 3 after fertilization. Furthermore, follicles with oocytes that produced embryos with higher scores on day 2 after fertilization had substantially higher levels of IGF-II, IGFBP-3, and IGFBP-4 than those arrested on the second day. In contrast, PAPP-A levels were significantly lower in follicles with early embryonic development than those with developmental arrest. Multiple regression analysis found that high combined levels of IGFBP-3 and IGFBP-4 in the FF, along with low levels of PAPP-A, were significantly correlated with increased fertilization rates and early embryonic development within the first 48 h after oocyte recovery. Conversely, high levels of IGFBP-1 and IGFBP-4 and low levels of IGF-I in the FF were associated with delayed embryonic development (between 48 and 72 h after oocyte recovery). Finally, significant stimulation of progesterone secretion in cultured GCs induced by recombinant IGF-II, IGFBP-3, and IGFBP-4 reinforced the functional role of these proteins in promoting late follicular development.

Data published by Moor et al. [49] suggest that the key to the maturation and viability of in vitro embryos lies within the compartment of follicular cells rather than the oocyte itself. Due to rapid changes associated with luteinization, cultured follicular cells likely fail to provide the maturing oocyte with the ordered set of instructive signals and nutrients necessary for acquiring developmental competence. Although much remains to be discovered regarding the nature, concentration, and transmission of these signals, it is already evident that various steroids, matrix metalloproteinases, and growth factors confer vitality to the maturing oocyte. Furthermore, the study suggests that significant improvements in the yield of viable embryos from in vitro matured oocytes could be achieved through systematic analysis of the somatic signals originating from the pre-ovulatory follicle. Thus, the oocyte depends on granulosa cells to supply essential nutrients and crucial regulatory signals during development. Granulosa cells must be adequately differentiated to initiate and transmit these signals to the oocyte effectively.

Barnes et al. [50] emphasized that oocytes matured in vitro from follicles in the early stages of atresia degeneration) show greater competence for embryonic development than those from actively growing follicles. This suggests that the acquisition of developmental capacity occurs before in vitro maturation and may be induced or favored by ovarian rest without gonadotropins in vivo or by ovarian incubation in vitro. It is plausible that the acquisition of developmental competence follows a common signaling or differentiation pathway involving both the oocyte and the surrounding granulosa cells, regardless of the final fate of the oocyte, whether ovulatory or degenerative. Early follicular atresia represents a distinctive observable characteristic in vitro, which is correlated with the increased developmental potential of oocytes, highlighting the significant influence of the follicular environment on the developmental competence of oocytes.

While various studies have reported significant bidirectional communication between the oocyte and granulosa cells, and despite the hypothesis of potential negative impacts resulting from early cumulus removal, the available clinical data are currently contradictory. Research conducted by Hassan et al. [51] aims to provide a clear perspective through a randomized prospective study conducted on sibling oocytes to evaluate the efficacy of pre-incubation with an intact cumulus in intracytoplasmic sperm injection (ICSI). Oocytes were randomly assigned to three distinct protocols: protocol A (immediate denudation followed by immediate injection), protocol B (delayed denudation followed by delayed injection), and protocol C (early denudation followed by delayed injection). The primary outcome measures followed by ICSI included oocyte maturation, fertilization capability, and cleavage rates. The results demonstrated that nuclear maturation, cytoplasmic maturation, and oolemma properties significantly benefited from the pre-incubation of oocytes with an intact cumulus before the ICSI procedure. Therefore, it was concluded that pre-incubation of the cumulus before ICSI improves treatment outcomes overall. It was also suggested that the specific duration of pre-incubation should be carefully considered and defined within the particular treatment protocol.

A study published by Alvares et al. [52] aimed to investigate the relationship between the cytoplasmic maturation of oocytes and their chromosomal status, examining how these characteristics influence the reproductive outcomes of patients undergoing assisted reproductive technologies (ART). Fragments of the first polar body were collected for chromosomal analysis using comparative genomic hybridization (aCGH). Oocytes were subjected to immunocytochemistry (ICC) to assess the levels of inactive maturation promoting factor (MPF) and the conformational alignment of the metaphase plate.

The results revealed that most mature MII oocytes exhibited a normal metaphase plate and were chromosomally normal. In contrast, immature oocytes showed a high frequency of abnormal metaphase plates, with only one-third being euploid. Among the unsuccessfully fertilized oocytes, 100% of the mature ones had a normal metaphase plate, with over 70% being euploid. However, less than 40% of immature oocytes displayed a normal metaphase plate, and only 50% were chromosomally normal. Overall, the rates of aneuploidy and metaphase plate anomalies in immature oocytes were significantly higher than in mature oocytes. Table 2 lists the factors that can influence cytoplasmic maturation.

## 4. Oocyte Maturation—Mitochondrial Maturation

Unlike the nucleus, mitochondria are the only organelles in animal cells that contain their genetic material, known as mitochondrial DNA (mtDNA). During oocyte maturation, there is a significant increase in the number of mtDNA copies and substantial changes in mitochondrial distribution [54]. Because oocyte maturation requires high ATP consumption to sustain continuous transcription and translation processes, it is crucial to ensure sufficient functional mitochondria. Pharmacological treatments and mitochondrial supplementation have been proposed to improve oocyte quality and enhance fertility [55], targeting both increased ATP production and reduced levels of reactive oxygen species (ROS) [56]. Additionally, recent research has highlighted the critical role of mitochondrial-derived metabolites in regulating epigenetic modifiers, providing a mechanistic basis for the crosstalk between mitochondria and the nucleus [54].

This interaction among subcellular compartments facilitates the modulation of gene expression in response to particular metabolic conditions, highlighting the significance of mitochondrial interactions in epigenetic regulation and developmental processes. Kirillova et al. [57] proposed a direct correlation between oocyte quality, mtDNA quantity, and ATP availability. Suboptimal conditions during in vitro maturation (IVM) may affect mitochondrial morphology and alter the expression of genes encoding proteins involved in mitochondrial function [58]. Mitochondria with compromised functionality exhibit a reduced capacity to neutralize reactive oxygen species (ROS), leading to oxidative stress. The idea has been hypothesized [57] that using antioxidants could improve mitochondrial function during oocyte maturation. Various categories of antioxidants have been explored in animal models and human oocytes subjected to IVM, yielding promising preliminary results. There is great hope for developing new IVM systems integrated with reagents targeting mitochondria to enhance oocyte developmental potential. In patients with reproductive disorders, quantitative defects, such as depletion, and qualitative defects, such as mutations, in mtDNA have been observed [59]. These defects suggest that mitochondrial deficiency may be correlated with oocyte maturation failure [60].

The study conducted by Raad et al. [61] highlighted that an increase in mtDNA levels in luteinized granulosa cells is associated with decreased cell viability, reduced first polar body [PBI] size, and increased PBI fragmentation in oocytes. This increased PBI fragmentation was linked to a significantly lower fertilization rate, suggesting that mtDNA levels in granulosa cells may negatively impact oocyte quality [62]. Pasquariello et al. [63] revealed that aged human oocytes exhibit reduced mitochondrial activity and decreased mitochondrial membrane potential, accompanied by increased mtDNA copies compared to younger oocytes. Maternal aging has been associated with mitochondrial dysfunction, which compromises oocyte quality and increases the risk of embryonic aneuploidies [64,65]. The integration of antioxidants during in vitro maturation has been shown to improve mitochondrial function and reduce oxidative stress, suggesting that the latter is a key factor in the decline of oocyte quality associated with advanced maternal age [66]. Several publications have already highlighted how mitochondrial replacement therapy (MRT) can enhance reproductive outcomes [67,68] by replacing dysfunctional mitochondria with healthy ones from donor oocytes, thereby rejuvenating the oocyte environment and improving developmental potential [69,70].

Mitochondrial transplantation represents a promising frontier in reproductive medicine, with the potential to improve oocyte quality and prevent the transmission of pathogenic mtDNA mutations [71,72]. However, mtDNA heteroplasmy remains a critical variable when assessing the effectiveness and outcomes of ooplasmic transfer [73,74]. The study conducted by Lan et al. [75] examined the interaction between oocytes and granulosa cells (CGCs) during oocyte maturation. The results indicate that the number of mtDNA copies in CGCs is synchronized with oocyte maturation and varies according to the maturation stage. Despite variations in mtDNA copy numbers among CGCs at different maturation stages, mitochondrial activity does not show significant changes. However, replication and transcription of mtDNA may influence each other, which could explain the discordance between changes in mtDNA copy numbers and mitochondrial gene expression levels. Novin et al. [76] revealed that during human oocyte maturation, the expression of the nuclear genes TFAM and NRF1 significantly correlates with the expression of the mitochondrial gene MT-CO1 at MI and MII stages, suggesting an increase in mitochondrial transcription. Conversely, such a correlation was not observed at the GV stage. These results indicate that ATP production during oocyte maturation is associated with the regulation of mtDNA. The study conducted by Bi et al. [77] employed an advanced technique to perform a detailed analysis of mtDNA in single human oocytes, highlighting the presence of rare mtDNA variants that could have significant implications for complex diseases. Clonal expansion of large structural variants of mtDNA was also demonstrated. These findings suggest potential improvements in pre-implantation diagnosis of mitochondrial diseases.

The paradox of preferential mtDNA replication describes the phenomenon whereby mutant variants of mitochondrial DNA are replicated and transmitted to a greater extent than their wild-type counterparts despite such mutations potentially compromising mitochondrial function and the organism as a whole. This behavior appears paradoxical, as natural selection should theoretically eliminate deleterious mutations. The study conducted by Zhang et al. [78] highlights that, in human oocytes, mutations such as m.8993T>G may favor the replication of mitochondria with high membrane potential. This increased potential may promote the replication and transmission of pathogenic mtDNA mutations. Mitochondrial DNA methylation has been confirmed as relevant for cellular function and linked to aging and diseases, but its role during oocyte maturation and early embryonic development remains unclear. Using bisulfite sequencing, Fan et al. [79] did not detect methylation in mtDNA during these stages in mice. The absence of methylation may favor mtDNA expression, supporting mitochondrial functions. Additionally, there is an emphasis on the need for more sensitive methods to study mtDNA methylation. Steffann et al. [80] investigated certain mtDNA point mutations that appear to undergo negative selection during female gametogenesis, with a critical threshold dependent on the specific mutation. Below this threshold, the presence of mtDNA mutations does not seem to affect oocyte maturation or early embryonic development, as demonstrated by a large sample of oocytes and embryos with high mutation frequencies. Table 3 lists the factors that can influence mitochondrial DNA modifications of oocytes

## 5. Maturation of Oocytes—Other Factors Influencing Maturation

According to several scientific reports [81,82], both oocyte maturation and activation during fertilization are regulated by variations in intracellular levels of Ca^2+^. The role of Ca^2+^ fluctuations during fertilization is well documented, as they are both necessary and sufficient for oocyte activation. However, the mechanism by which sperm induce Ca^2+^ variations during fertilization and how different Ca^2+^ fluctuation patterns influence embryonic development is not fully understood. The role of Ca^2+^ in activating oocyte maturation is less defined, although it is known that inhibiting intracellular Ca^2+^ fluctuations can prevent meiotic maturation at specific stages. Ullah et al. [82] proposed a mathematical model to identify critical factors determining the differentiation of the Ca^2+^ signaling during oocyte maturation. The results indicate that increasing the affinity of the IP3 receptor (IP3R) replicates both the elementary and global dynamics of Ca^2+^ observed experimentally after oocyte maturation.

Moreover, increasing the affinity for IP3R, due to the system’s dependency on both SERCA (sarcoplasmic reticulum Ca^2+^-ATPase) and IP3R, shifts the equilibrium of the system towards a new steady state characterized by elevated cytosolic Ca^2+^ levels. This state is essential for facilitating fertilization. Therefore, the model provides a unique perspective on how even small modifications in the fundamental molecular mechanisms of Ca^2+^ signaling components can significantly influence the spatiotemporal properties of intracellular Ca^2+^ dynamics in oocytes.

Battaglia et al. [83] investigated another aspect related to oocyte maturation, specifically the distribution of microtubule organizing centers (MTOC) in human oocytes using taxol, a drug that promotes microtubule polymerization. Oocytes obtained from patients undergoing assisted reproductive technology (ART) were examined during different stages of meiotic maturation using confocal fluorescence microscopy.

During the prophase of meiosis I, taxol did not induce microtubule nucleation in any part of the cell, with only a few microtubules in the oocyte cortex. Upon transitioning from prophase to metaphase, during the breakdown of the germinal vesicle, taxol stimulated the formation of a limited number of aster-like microtubule arrays in the cortex, primarily near the nucleus. Taxol-treated oocytes at metaphase I exhibited a significant number of aster-like microtubule arrays predominantly located in the cortical region of the oocyte, with some smaller arrays visible in the endoplasmic areas. No increase in the density of asters was noted in specific cortical or endoplasmic regions. During metaphase II of meiosis, taxol-treated oocytes exhibited a similar response to that observed during metaphase I. Again, the microtubule arrays were predominantly distributed in the cortex, with less dense asters in the endoplasm. Taxol also affected the mitotic spindle during metaphase, increasing the density and hyperelongation of microtubules at the spindle poles compared to untreated oocytes. The metaphase chromosome plate was significantly altered by taxol treatment, likely due to the forces generated by the elongation of the microtubules. Human oocytes [83] develop microtubule organizing centers (MTOC) as meiotic maturation progresses beyond the block in prophase I. The first MTOCs are situated perinuclearly, but their number and distribution significantly increase when the oocyte enters the metaphase. It is hypothesized that the human centrosome recruits various MTOC domains to assemble the meiotic spindles during both meiotic divisions. Additionally, one or more MTOCs not associated with the spindle may combine with sperm centrosomal material during fertilization to form the complete centrosome required for embryonic mitosis. The widespread diffusion of MTOCs in the cortex may ensure this recombination, regardless of the point of the sperm’s incorporation into the oocyte.

Prorenin [PR] is present in high concentrations in the follicular fluid (FF) of preovulatory follicles and represents the predominant form of renin in this context. Gonadotropins regulate the biosynthesis and secretion of PR in the ovary. In the study published by Itskovitz et al. [84], PR and steroid levels were measured in FF samples taken from 136 follicles. Follicular fluids were obtained 36 h after hCG injection from 41 patients who were stimulated with gonadotropins and underwent follicular puncture and oocyte recovery for ART. PR levels in FF were correlated with gonadotropin levels and the maturation stage of the oocyte-cumulus complex. The average PR levels in 62 FF samples containing mature oocytes were 2620 ± 157 ng/mL.h (728 ± 44 ng/L.s; range, 1020–6880 ng/mL.h, 283–1911 ng/L.s). A subset of sixteen of these follicles, containing mature oocytes from seven women who achieved conception, had PR levels confined to the lower range of 1030–2720 ng/mL.h (286–756 ng/L.s). No patient conceived with PR levels in FF exceeding 2800 ng/mL.h (778 ng/L.s), although one-third of all mature follicles had PR levels above this range. Lower PR levels were found in FF samples containing immature oocytes [germinal vesicle stage] associated with compact (1665 ± 480 ng/mL.h; 463 ± 133 ng/L.s; n = 22; *p* < 0.02) or expanded (1785 ± 193 ng/mL.h; 496 ± 54 ng/L.s; n = 24; *p* < 0.005) cumuli. A subgroup (n = 5) of follicles with immature oocytes and compact cumuli had very high levels of PR in FF, ranging from 3830–7520 ng/mL.h (1064–2089 ng/L.s), while the rest had levels below 1300 ng/mL.h (361 ng/L.s). Progesterone and estradiol (E2) levels were lower in FF associated with compact (*p* < 0.005) or expanded (*p* < 0.02) cumuli containing immature oocytes compared to those containing mature oocytes. Testosterone (T) and androstenedione levels were measured in only a fraction of samples, and no significant differences emerged between follicles containing mature and immature oocytes. However, T and androstenedione were elevated in the subgroup of follicles with immature oocytes and very high levels of PR. Among the hormones measured, T showed the most significant relationship with PR (r = 0.62; n = 49; *p* < 0.001).

Another study by Llonch et al. [85] examined the effect of age and body mass index (BMI) on oocyte quality at the transcriptome level using single-cell RNA sequencing with the Smart-seq2 protocol. The results showed that the maturation stage of the oocyte is the primary factor of transcriptomic variability, with age having a greater impact on in vitro matured oocytes (IVM-MII) than GV oocytes. Changes were identified in transcripts related to oxidative stress, mitochondrial function, and chromosomal segregation. Network analysis revealed potential key regulators such as basonuclin 1 (BNC1), a transcription factor that regulates rRNA transcription, and SON, an RNA-binding protein that promotes pre-mRNA splicing.

The study conducted by Buratini et al. [86] investigated the regulation of gene expression governing the ovulatory cascade and the formation of the extracellular matrix in cumulus cells. The results demonstrated that factors secreted by the oocyte suppress the expression of prostaglandin synthase-2 (PTGS2), a key promoter of maturation in cumulus cells. The suppression of PTGS2 by factors secreted from the oocyte appears to reflect a mechanism by which the oocyte controls the timing of its maturation, preventing premature meiotic resumption and thus promoting optimal synchronization between nuclear and cytoplasmic maturation and adequate embryonic competence.

A 2024 [87] review explores how mechanistic studies conducted in vitro offer crucial insights into the significant effects of bisphenol exposure on oocyte health. Bisphenols can interact directly with receptors in either an agonistic or antagonistic manner, disrupting receptor signaling pathways and hormone production. Research has shown that BPA and its analogues, such as BPS, BPB, BPF, and BPAF, exhibit comparable or even stronger estrogenic activity on human estrogen receptors α and β. Additionally, many bisphenols have similar effects on other receptors, including the androgen receptor, pregnane X receptor, constitutive androstane receptor, and glucocorticoid receptor. This functional similarity between BPA and its alternatives helps explain the consistent impact they have on oocytes and follicles, highlighting the pressing need to reassess their safety thresholds. Beyond their direct actions on receptors, bisphenols can also indirectly affect the female reproductive system by triggering apoptosis, oxidative stress, and inflammation in various ovarian cells. Numerous in vitro studies have demonstrated that exposure to BPA or its alternatives leads to oxidative stress and, in some cases, apoptosis, which are key factors affecting oocyte health. These alterations in oxidative balance and apoptotic regulation, observed in cultured neonatal ovaries, pre-antral follicles, cumulus-oocyte complexes, and oocytes, play a major role in understanding how bisphenols impair follicle and oocyte development and maturation, ultimately compromising oocyte quality.

Propylparaben (PrPB) is a recognized endocrine-disrupting chemical widely used as a preservative in pharmaceuticals, food products, and cosmetics. It has been detected in human urine and serum, and its exposure has been linked to functional impairments in reproductive health. However, the specific effects of PrPB on mammalian oocytes remain largely unexplored. In the present study, these findings demonstrate that PrPB exposure interferes with mouse oocyte maturation in vitro, leading to meiotic resumption arrest and failure in the extrusion of the first polar body. Exposure to 600 μM PrPB significantly reduced the rate of germinal vesicle breakdown (GVBD) in oocytes. Further investigation revealed that PrPB induced mitochondrial dysfunction and oxidative stress, which subsequently caused DNA damage in the oocytes. This damage disrupted the activity of the maturation-promoting factor (MPF) complex, specifically Cyclin B1 and Cyclin-dependent kinase 1 (CDK1), leading to a G2/M phase arrest. Additional experiments indicated that PrPB exposure resulted in aberrant spindle morphology and chromosome misalignment, which was attributed to destabilized microtubules. Furthermore, PrPB impaired the attachment between microtubules and the kinetochore, triggering sustained activation of BUB3 and BubR1, two critical spindle-assembly checkpoint (SAC) proteins. Collectively, these findings suggest that PrPB disrupts mouse oocyte maturation by interfering with the MPF-regulated G2/M transition and the SAC-dependent metaphase-to-anaphase transition [88].

A recent meta-analysis suggests that to overcome oocyte maturation arrest, the use of a dual trigger, combining GnRH agonist and hCG, should be considered [89]. This approach has been shown to significantly improve the number of oocytes retrieved and their maturation, leading to better outcomes in in vitro fertilization (IVF) cycles, especially in fresh embryo transfer (ET) cycles. Dual triggering, when compared to the traditional hCG-only trigger, results in a higher number of mature oocytes and addresses the challenge of oocyte maturation arrest. The improvement is likely due to the synergistic effect of GnRH agonists, which induce an LH and FSH surge, and hCG, which supports further maturation. Additionally, enhanced endometrial receptivity contributes to better clinical pregnancy rates and a higher live birth rate (LBR), particularly in fresh ET cycles. However, the beneficial effects were not observed in frozen-thawed ET. Importantly, dual triggering did not increase miscarriage rates or ovarian hyperstimulation syndrome (OHSS). These findings suggest that dual triggering offers a promising strategy to overcome oocyte maturation arrest and improve IVF outcomes, particularly in fresh ET cycles, though further research is needed to optimize protocols and minimize risks. A novel publication discusses the challenges and potential of in vitro maturation (IVM) for fertility preservation [90]. Limitations include low recovery rates of oocytes and limited maturation, especially from small antral follicles. Double-IVM cycles and combining IVM with ovarian tissue cryopreservation (OTC) are suggested to increase mature oocytes. Biphasic IVM, which prevents premature maturation, shows promise in improving oocyte quality and safety concerns regarding epigenetic changes in IVM oocytes are minimal.

A recent review addresses Empty Follicle Syndrome (EFS) in assisted reproductive technology, presenting it as a subtype of Oocyte Maturation Abnormalities (OMAs) with significant genetic involvement [91]. EFS includes genetic EFS (gEFS), where mature oocytes are absent despite proper hCG levels, and functional EFS (fEFS), often due to issues with hCG administration. The study examined 17 women with gEFS, identifying mutations in genes such as FSHR, LHCGR, TACR3, ZP1, and ZP3, although some cases showed no detectable mutations.

Clinical trials of in vitro maturation (IVM) protocols, including letrozole-primed IVM with growth hormone, indicated that while oocytes and embryos could occasionally be obtained, successful live births were infrequent. Mutations in ZP1 and LHCGR were linked to varied EFS manifestations, including biochemical pregnancies and zona-free oocytes. The review emphasizes that EFS likely involves immature, not truly empty, follicles and suggests that genetic testing through whole-exome sequencing could improve diagnosis and management.

In a recent publication [92], researchers analyzed whole-exome sequencing data from 1024 women experiencing oocyte maturation arrest or showing degenerated and morphologically abnormal oocytes, compared to 2868 healthy controls, to conduct extensive population-level and gene-focused burden assessments on mitochondrial genes. This approach identified cytochrome c oxidase assembly protein 15 (COX15) as a key candidate gene. The study demonstrated that biallelic pathogenic variants in COX15 trigger ferroptosis in human oocytes, causing female infertility through a recessive inheritance mechanism. Functional analyses revealed that COX15 variants impair mitochondrial respiration in yeast, Saccharomyces cerevisiae, and reduce COX15 protein levels in HeLa cells. Additionally, conditional deletion of Cox15 in oocytes led to disrupted homeostasis of Fe^2+^ and reactive oxygen species, resulting in mitochondrial dysfunction and increased oocyte sensitivity to ferroptosis. Importantly, treatment with ferrostatin-1, a ferroptosis inhibitor, was able to reverse the ferroptotic phenotype in oocytes both in vitro and ex vivo. These findings not only provide a genetic diagnostic marker for oocyte development defects but also broaden the spectrum of mitochondrial disorders linked to female infertility, offering unique insights into the role of ferroptosis in human oocyte abnormalities.

## 6. Materials and Methods

In this review, we identified studies describing oocyte maturation at both the nuclear and cytoplasmic levels, as well as other factors involved in activation processes. We searched PubMed, Scopus, ResearchGate, Web of Science, and preprint archives for articles published up to October 2024, using the following search terms: nuclear oocyte maturation, cytoplasmic oocyte maturation, factors involved in cytoplasmic maturation, effect of mitochondria on cytoplasmic maturation. Four independent authors reviewed the search results to exclude studies that did not fall within the scope of our research.

## 7. Conclusions

**Figure 1:**
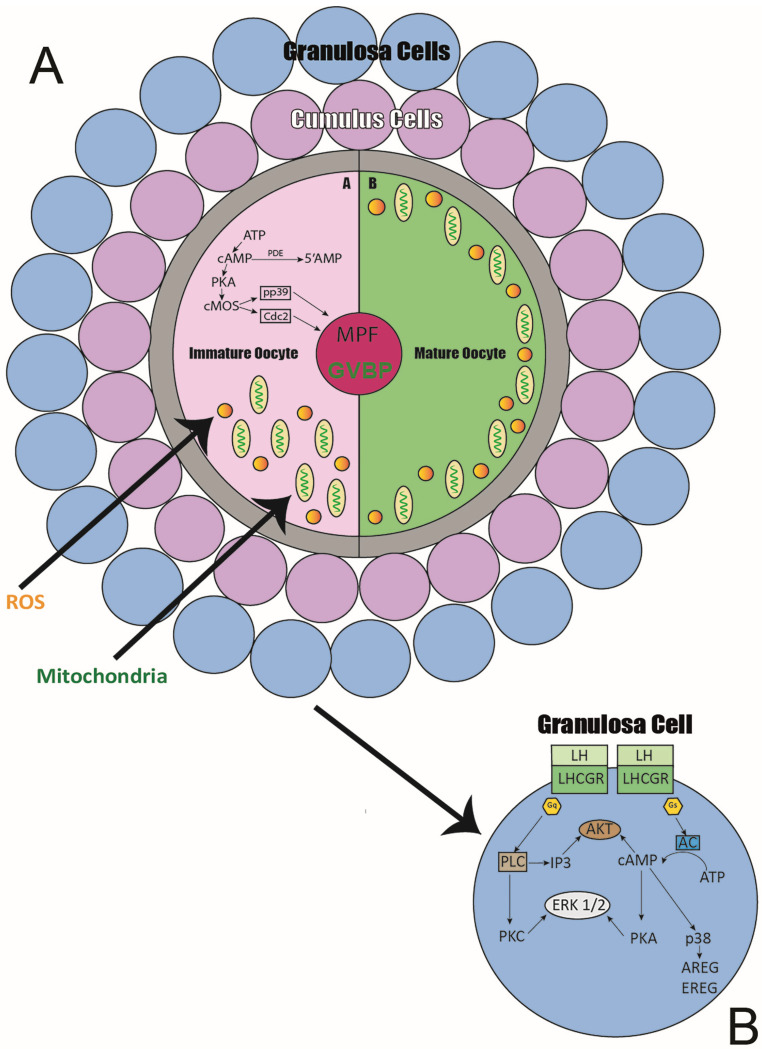
The image illustrates key processes in oocyte maturation. (**A**) Description of the pathways activating cytoplasmic and nuclear maturation, along with mitochondrial redistribution toward the cortical cytoplasm. (**B**) Highlights the granulosa cell pathways that regulate and support this maturation.

In conclusion, oocyte maturation is a multifaceted process shaped by numerous factors, including mitochondrial activity, calcium dynamics, and the interactions between cumulus cells and oocytes. Investigations have demonstrated that oocyte quality is essential for the effectiveness of assisted reproductive technologies, carrying profound implications for female fertility. The image below illustrates the key biological processes involved in oocyte maturation. Pathways responsible for the activation of both cytoplasmic and nuclear maturation are depicted, along with the redistribution of mitochondria toward the cortical areas of the cytoplasm (Figure 1A). Subsequently, the focus shifts to the pathways facilitated by granulosa cells, which play a crucial role in supporting and regulating the maturation process (Figure 1B). This comprehensive view highlights the coordinated actions within the oocyte and its surrounding cellular environment.

Confronting defects in oocyte maturation represents a significant challenge and highlights important research areas for geneticists and clinicians. We have dedicated a publication to this topic to thoroughly examine the complexities related to oocyte maturation and to deepen our understanding of its intricate processes [92,93]. While oocyte donation may represent the most straightforward pathway to achieving pregnancy, it may not be a feasible option for all couples. Therefore, further investigation into the underlying causes of these maturation defects is essential. This should include comprehensive evaluations of oocytes using electron and fluorescent microscopy, as well as the analysis of follicular fluid to identify key factors involved in maturation, such as cytokines, cell adhesion proteins, and other local signals that may influence the meiotic process. Future investigations must endeavor to elucidate the mechanisms governing intracellular calcium dynamics and their ramifications on oocyte maturation. Moreover, probing the interplay between mitochondrial functionality and oocyte quality could yield innovative strategies for the enhancement of fertility treatments. The adoption of advanced methodologies, such as genetic editing and epigenetic analysis, promises to enrich our comprehension and management of reproductive health. Despite the intricate challenges associated with maturation defects, there exists the potential for certain women to attain pregnancies utilizing autologous gametes and IVM, thereby underscoring the imperative for continued inquiry in this domain.

## Figures and Tables

**Table 1:** Factors affecting cytoplasmic maturation of oocytes.

Factor	Description
cAMP (Cyclic Adenosine Monophosphate)	The decrease in intracellular cAMP levels is associated with the resumption of meiosis in the oocyte [28].
PDE (Phosphodiesterase)	An enzyme that degrades cyclic nucleotides such as cAMP, regulating the resumption of meiosis [29].
PKA (Protein Kinase A)	Kinase that regulates oocyte maturation; its inactivation is necessary for GVBD [32,36].
PKG (Protein Kinase G)	Involved in the signaling cascade that regulates meiosis; activated by cGMP [33].
PKC (Protein Kinase C)	Calcium-activated kinase involved in the regulation of oocyte maturation [33].
cGMP (Cyclic Guanosine Monophosphate)	Molecule that, through the regulation of cAMP, participates in the control of oocyte maturation [27].
MPF (Maturation-Promoting Factor)	A kinase complex that promotes oocyte maturation and progression through meiosis [24].
C-mos	Oocyte-specific kinase essential for the activation of MPF during maturation [24].
AC (Adenylate Cyclase C)	An enzyme that catalyzes the conversion of ATP to cAMP, activated by the pre-ovulatory LH surge [35].
VDBP (Vitamin D Binding Protein)	A protein whose role decreases during nuclear maturation in response to the LH surge [35].
Gap Junctions	Cellular connections that break down during maturation, reducing the transfer of cGMP [35].
p34cdc2 Kinase	A subunit of MPF involved in the control of the meiotic cell cycle [24].
Cyclin B	A regulatory subunit of MPF, whose activity is necessary for meiotic progression [24].

**Table 2:** Factors affecting cytoplasmic maturation of oocytes.

Factor	Description
cAMP/cGMP regulated by CNP	Low and non-variable CNP levels during the final maturation of the follicle. GDF9 and BMP15 are present in very low concentrations [38,39].
EGF-related peptides (AREG, EREG)	AREG is upregulated during the first 12–17 h of follicular maturation. EGF and IGF-I enhance in vitro oocyte maturation. However, a 6 h incubation with EGF does not affect the cytoplasmic maturation of oocytes obtained after treatment [38,40,45,46].
Growth factors (GDF9, BMP15, MDK)	MDK is upregulated during the first 12–17 h, whereas GDF9 and BMP15 show low influence in regulating oocyte maturation in vivo [38,41,44].
Meiosis-activating sterol (FF-MAS)	Genes regulating the synthesis and metabolism of FF-MAS are significantly controlled to favor accumulation during the first 12–17 h of follicular maturation [42,43].
Insulin-like growth factors (IGF)	Elevated concentrations of IGFBP-1 are correlated with mature oocytes and high embryonic quality. IGF-II, IGFBP-3, and IGFBP-4 are associated with better early embryonic development; PAPP-A is linked to late embryonic development [47,48].
Bidirectional communication oocyte-granulosa	The oocyte relies on granulosa cells for essential nutrients and regulatory signals. Preincubation of oocytes with intact cumulus before ICSI improves treatment outcomes [49,50,53].
Oocyte developmental competence	In vitro matured oocytes from atretic follicles show greater competence for embryonic development compared to those from actively growing follicles [49,51].
Cytoplasmic and chromosomal status of oocytes	Immature oocytes show a high frequency of chromosomal abnormalities and metaphase plate defects compared to mature oocytes [51].

**Table 3:** Factors affecting mitochondrial DNA modifications of oocytes.

Factor	Description
Increase of mtDNA during oocyte maturation	The increase in mtDNA copies and mitochondrial distribution are crucial for oocyte maturation, supporting ATP production [52].
Improvement of oocyte quality with mitochondrial treatments	Targeted treatments and supplements aimed at mitochondria can improve oocyte quality by increasing ATP and reducing oxidative stress [52].
Interaction between mitochondria and nucleus in epigenetic regulation	Mitochondrial metabolites influence epigenetic modifiers, adjusting gene expression to metabolic needs [52].
Correlation between mtDNA, ATP, and oocyte quality	Oocyte quality depends on the amount of mtDNA and ATP; suboptimal in vitro conditions can damage mitochondrial function [54].
Antioxidants and improvement of mitochondrial function	Antioxidants can reduce oxidative stress, improving oocyte quality during maturation [54].
mtDNA defects and failure of oocyte maturation	Quantitative or qualitative mtDNA defects are associated with oocyte maturation failure in reproductive disorders [54].
Increase of mtDNA and decrease of oocyte quality	An excess of mtDNA in granulosa cells is correlated with lower oocyte quality and reduced fertility [55].
Mitochondrial dysfunction in aged oocytes	In aged oocytes, there is reduced mitochondrial activity and increased mtDNA copies, linked to a decline in oocyte quality [56].
Relationship between oocytes and granulosa cells	In aged oocytes, reduced mitochondrial activity and an increase in mtDNA copies are observed, which are associated with a decline in oocyte quality [56].
Correlation between nuclear and mitochondrial genes	During maturation, nuclear and mitochondrial genes show correlated expression, indicating a coordinated regulation of ATP production [58].
Rare mtDNA variants and pre-implantation diagnosis	Rare mtDNA variants in oocytes can affect the pre-implantation diagnosis of mitochondrial diseases [59].
Preferential replication of mtDNA mutations	Some mtDNA mutations promote the replication of mitochondria with high membrane potential, leading to the transmission of pathogenic mutations [60].
Role of mtDNA methylation	mtDNA methylation, not detected during oocyte maturation, could influence gene expression and aging [61].
Selection of mtDNA mutations during gametogenesis	Some mtDNA mutations undergo negative selection during gametogenesis, affecting oocyte maturation at critical levels [62].

## Data Availability

Data are contained within the article.
